# EZH2 Expression in Head-and-Neck Squamous Cell Cancer in Young Patients

**DOI:** 10.3390/ijms25105250

**Published:** 2024-05-11

**Authors:** Mónika Révész, Ferenc Oberna, András Slezák, Erika Tóth, Örs Ferenczi, István Kenessey, Zoltán Takácsi-Nagy

**Affiliations:** 1 Multidisciplinary Head and Neck Cancer Center, National Institute of Oncology, H-1122 Budapest, Hungary; oberna.ferenc@oncol.hu; 2 Center of Tumor Pathology, National Institute of Oncology, H-1122 Budapest, Hungary; slezak.andras@oncol.hu (A.S.); dr.toth.erika@oncol.hu (E.T.); 3 Center of Radiotherapy, National Institute of Oncology, H-1122 Budapest, Hungary; ferenczi.ors@oncol.hu; 4 Hungarian Cancer Registry, National Institute of Oncology, H-1122 Budapest, Hungary; kenessey.istvan@oncol.hu; 5 Department of Pathology, Forensic and Insurance Medicine, Semmelweis University, H-1091 Budapest, Hungary; 6 National Tumor Laboratory Project, H-1122 Budapest, Hungary; 7 Department of Oncology, Semmelweis University, H-1083 Budapest, Hungary

**Keywords:** EZH2, young adults, head-and-neck cancer, prognostic factors

## Abstract

EZH2 (Enhancer of zeste homolog 2) promotes tumor growth and survival through numerous mechanisms and is a promising target for novel therapeutic approaches. We aimed to characterize the expression of EZH2 in the tumors of young head-and-neck squamous cell cancer (HNSCC) patients in comparison with the general HNSCC patient population. We used formalin-fixed, paraffin-embedded tissue blocks from 68 random young HNSCC patients (≤39 years, median age: 36 years; diagnosed between 2000 and 2018), which were compared with the samples of 58 age- and gender-matched general HNSCC subjects (median age: 62 years; all diagnosed in the year 2014). EZH2 and p53 expression of the tumors was detected using immunohistochemical staining. Lower EZH2 expression was found to be characteristic of the tumors of young HNSCC patients as opposed to the general population (median EZH2 staining intensity: 1 vs. 1.5 respectively, *p* < 0.001; median fraction of EZH2 positive tumor cells: 40% vs. 60%, respectively, *p* = 0.003, Mann–Whitney). Cox analysis identified a more advanced T status (T3-4 vs. T1-2), a positive nodal status, and alcohol consumption, but neither intratumoral EZH2 nor p53 were identified as predictors of mortality in the young patient group. The lower EZH2 expression of young HNSCC patients’ tumors discourages speculations of a more malignant phenotype of early-onset tumors and suggests the dominant role of patient characteristics. Furthermore, our results might indicate the possibility of an altered efficacy of the novel anti-EZH2 therapies in this patient subgroup.

## 1. Introduction

Head-and-neck squamous cell carcinoma (HNSCC) is the sixth-most-common cancer worldwide, with approximately 880,000 new cases and 445,000 deaths annually [1,2]. Although it usually affects patients over 60 years of age, the number of young patients is increasing [3] (it is now <39 years of age according to the National Comprehensive Cancer Network (NCCN) Adolescent and Young Adult (AYA) Oncology guidelines [4]). The causes of this trend are yet to be clarified [5], but current evidence makes the exclusive role of any single factor unlikely. Although smoking and alcohol consumption are well-defined risk factors for HNSCC, the cumulative doses are lower in young individuals. We previously found the prevalence of tobacco and alcohol consumption among young HNSCC patients to be between that of the general regional population (>15 years) and the general HNSCC patient population [6]. Furthermore, the age distribution of young patients was heavily distorted towards the cut-off value of 39 years, and the oral cavity—the site with the most environmental exposure—was the most common location among the young but not the general patient population. Our pilot results suggested that young HNSCC patients might have greater vulnerability to various carcinogens [6]. This might originate either from defective cell cycle control or the dysfunctional clearance of malignant cells. In the latter scenario, tumor cells with a less aggressive phenotype might also survive, which might theoretically be in line with the more favorable survival data for young patients [6,7].

**Figure 1:**
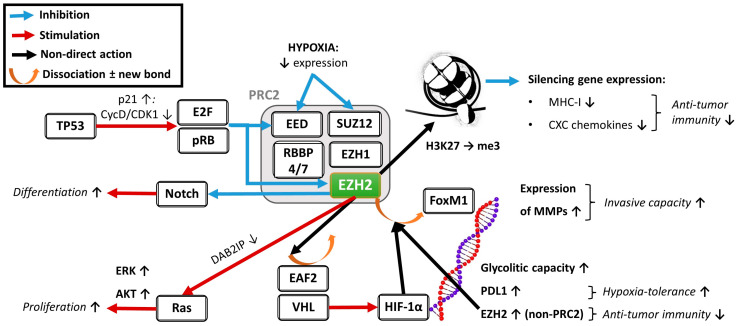
Summary of the main molecular interactions (with functional outcomes italicized) of EZH2. EZH2 might act both as a PRC2 member and independently through interactions with other factors. Hypoxia, a common phenomenon in the tumor microenvironment, might up-regulate the PRC2-independent EZH2 functions both by down-regulating the expression of PRC2 members (EED and SUZ12) and by facilitating the binding of free EZH2 to FoxM1 instead of other PRC2 members, thus modulating the expression of MMPS, hence the invasive capacity of tumor cells. EZH2 itself might facilitate the dissociation of EAF2 from VHL, thus activating HIF-1α-linked transcriptional alterations, including the up-regulation of glycolitic capacity, hypoxia-tolerance, and the expression of free EZH2. The PRC2-linked effects of EZH2 also support cellular proliferation and might hinder differentiation. The tumor suppressor TP53 acts against the expression of PRC2 members (EED and EZH2) [10,11,12,13,14,15,16,17].

Multiple mechanisms may affect common pathways, amongst which epigenetic mechanisms play versatile roles in modulating cell proliferation, apoptosis inhibition, and tumor progression [8]. The enhancer of zeste homolog 2 (EZH2) seems to form an outstanding hub of cellular pathways promoting tumor viability. It is involved in regulating cellular survival (via enhancing both proliferation and hypoxia-tolerance), the immunogenicity of tumor cells (through the down-regulation of MHC Class I antigen presentation, hence the immunological visibility [9]), and the capacity for metastasis formation (through modulating interactions with the extracellular matrix) [10,11,12,13,14,15,16,17]. EZH2 exerts its functions both as an enzimatic catalytic subunit of polycomb repressive complex 2 (PRC2), chiefly through regulating genome structure and gene expression through the trimethylation of Lys-27 in histone 3 (H3K27me3), and also PRC2-independently by modulating further mediators, including the hypoxia-inducible factor-1α (HIF-1α) and FoxM1 [10,11,12,13,14,15,16,17]. Although reviewing all known functions of EZH2 would far exceed the scope of this work, we summarized its main functions associated with tumor cell viability and survival, as shown in Figure 1. EZH2 expression was identified as a negative prognostic marker in multiple solid tumors, including breast, lung, and colorectal cancers [18,19,20] and also HNSCC [21]. 

The intratumoral expression of the major tumor suppressor protein p53 detected via immunohistochemistry was reported to affect the prognosis of HNSCC patients (Wang et al. [22]); furthermore, it might inhibit the actions of EZH2 through promoting the maintenance of the pRB-mediated inhibition of E2F, hence the decreased expression and activity of PRC2 members (Kulaberoglu et al. [17]), and possibly also in a more direct way by inhibiting EZH2 expression through repressing the EZH2 gene promoter (Tang et al. [23]). Due to the profound roles of EZH2 and p53 in determining the aggressivity and prognosis of HNSCC, we aimed to compare their expression in young patients and the general HNSCC patient population to further characterize the nature of this greater risk that might result in early disease onset.

## 2. Results

### 2.1. Epidemiological Characteristics

**Figure 2:**
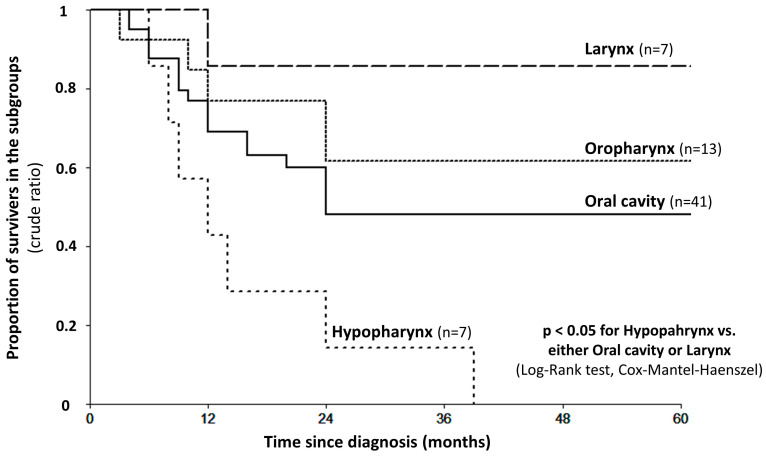
Five-year overall survival curves of subtypes of head-and-neck cancers in young patients.

The median age at diagnosis was 36 [total range 21–39] in the young and 62 [total range 45–88] in the general HNSCC group. Young patients with laryngeal cancer had the best five-year disease-free survival (DFS), amounting to 83.3%, followed by those with oropharyngeal, lip and oral cavity, and hypopharyngeal tumors (61.5%, 43.6%, and 0%, respectively). Of note, the general HNSCC (control) group is not epidemiologically representative, as its members were selected to match the young group for gender distribution and tumor localization (lip, oral cavity, mesopharynx, hypopharynx, or larynx). Group characteristics are shown in detail in Table 1. The survival data for the young HNSCC group are also presented in Figure 2.

### 2.2. Expression of EZH2 in Squamous Cell Carcinoma of the Head-and-Neck Region

**Figure 3:**
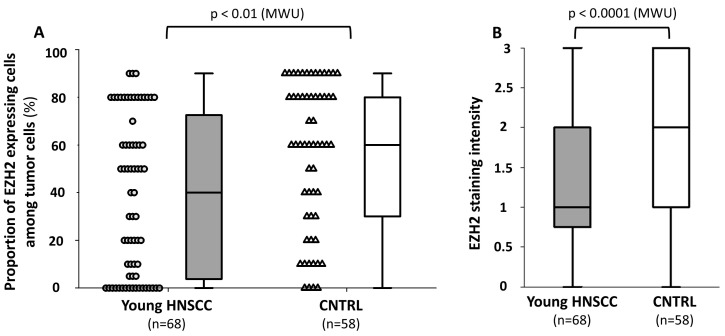
(**A**) Comparison of the median proportion of EZH2-expressing cells in the two examined groups, dots represent individual data points. (**B**) comparison of the median staining intensity of EZH2 (young and control groups). Medians, interquartile ranges and total ranges are indicated.

**Figure 4:**
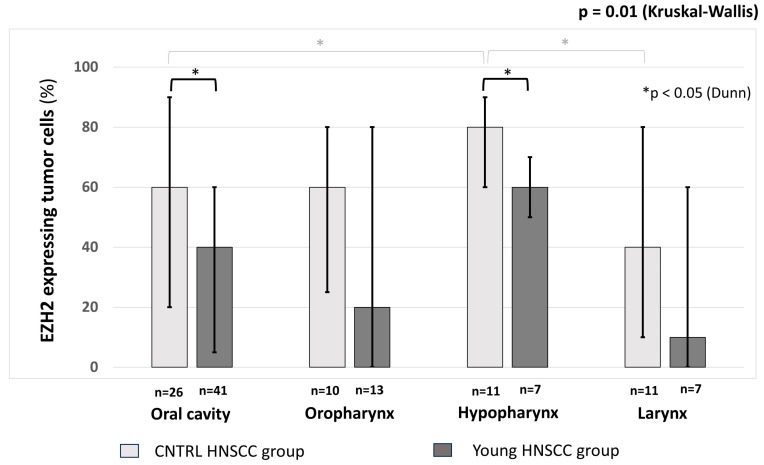
Proportions of EZH2-expressing tumor cells in certain locations in the young and control HNSCC groups. Medians and interquartile ranges are indicated.

EZH2 staining were predominantly nuclear. The median proportion of EZH2-expressing cells among all malignant cells was 60% [IQR: 30–80] in the general population and 40% [IQR: 3.75–72.5] in the young HNSCC group (*p* = 0.003; MWU, stat. power: 95.2%, Figure 3A). We also found a significant difference in the median staining intensity of EZH2 (young and control group medians: 1 [IQR: 0.75–2] vs. 1.5 [IQR: 1–2], respectively, *p* = 0.0001, MWU, Figure 3B). In the subgroup analysis, lower EZH2 expression both regarding the proportion of expressing cells and the intensity of staining was characteristic of young patients in all tumor localizations, although the difference only reached statistical significance for the oral cavity and hypopharynx when considering proportions (Figure 4). All EZH2 and p53 expression data are presented in Table 2. EZH2 expression was not correlated with histological grade, primary tumor status, or the presence of nodal metastasis. Higher EZH2 expression seems to characterize hypopharyngeal tumors when compared to either oral cavity or laryngeal malignancies (percentage of positive cells, control group; oral cavity 60% [IQR: 20–90] or larynx 40% [IQR: 10–80] vs. hypopharynx 80% [IQR: 60–90]; Dunn’s post hoc *p* < 0.05). Although a similar trend was observed for the young group, it did not reach statistical significance.

### 2.3. Survival Characteristics and Potential Associations

The five-year overall survival (OS) for all head-and-neck sites was 46.1% for young patients and 34.5% for the control group (*p* > 0.05, χ^2^). The 5-year OS was significantly better for patients with N0 vs. N+ nodal status in both groups (young group: 69% vs. 31.8%, *p* = 0.004, χ^2^; control group: 46.8% vs. 19.2%, *p* = 0.0074, χ^2^). A linear regression analysis suggested that there was only a marginal effect of the proportion of EZH2-expressing tumor cells on the crude disease-free survival of young patients, with borderline significance (F(1,66) = 3.95, *p* = 0.05, with an R^2^ = 0.06 and a regression coefficient of −0.4); thus, the one percent increase in the proportion of EZH2-expressing cells might be related to a 0.4-month decrease in the predicted DFS with great scatter in the data. Cox regression analysis was performed to test the true predictive values of tumoral EZH2 and p53 expression, alcohol and tobacco consumption, and T and N statuses (Table 3). Univariate Cox analysis identified a more advanced T status (T3-4 vs. T1-2), a positive nodal status, and alcohol consumption above the WHO medium risk category as significant predictors of mortality. In multivariate Cox analysis, the nodal status was verified as the single highly significant predictor of mortality, whereas alcohol consumption only reached borderline significance. Neither a higher proportion of EZH2-expressing tumor cells (defined as exceeding the group median of 40%) nor intratumoral p53 expression were identified as significant predictors of an unfavorable outcome among young HNSCC patients (although the trend of the HR was coherent with the known biological nature of both mediators).

We need to underline that the control group is not representative of the general patient population as it was selected to match the tumor site and gender distribution of the young study group.

## 3. Discussion

Here, we report that young HNSCC patients might be characterized by lower EZH2 and similar p53 expression when compared to the general HNSCC patient population. To the best of our knowledge, our study is the first to examine EZH2 expression in this subgroup. Our previous results suggested that the early onset of HNSCC might originate from a greater vulnerability to environmental agents [6]. Either diminished damage control at the cellular level or a weaker capacity of the immune system to eliminate tumor cells might be responsible [24,25,26]. EZH2 has a well-defined oncogenic role in cancer initiation, progression, metastasis, metabolism, and drug resistance and in the modulation of anti-tumor immunity in cancers of the prostate, breast, bladder, and esophagus, as well as gastric and non-small-cell lung carcinoma and lymphomas [18,27,28,29,30,31]. Recent studies suggested that the carcinogenesis of HNSCC is also driven by changes in epigenetic modifications [32] affecting the expression of tumor suppressor genes and oncogenes. EZH2 expression was found to be higher in HNSCC tumor cells than in corresponding non-tumoral regions, suggesting that an increased number of EZH2-positive cells might reflect increased cell proliferative activity [33,34]. Due to these results, EZH2 was defined as an excellent marker of the tumor aggressivity and tumorigenic potential of cancers [35]. In a recent paper, Wu et al. showed that EZH2 is a survival-related biomarker and associated with poor prognosis among HNSCC patients [36].

The lower EZH2 expression of young patients’ tumors discourages speculations on more-viable tumors (either through more successful immune evasion or a more malignant cellular phenotype) and draws our attention toward patient characteristics that might facilitate the survival of malignant cells. The lower EZH2 expression in young HNSCC patients might also highlight the clinical significance of personalized oncological therapies. Approximately two thirds of head-and-neck cancer patients present with locoregionally advanced stage disease, which requires aggressive multimodality therapy [37]. Ongoing studies are exploring more effective and tolerable targeted therapies to improve clinical outcomes. Recently, numerous EZH2 inhibitors have been introduced. Tazemetostat was the first EZH2 inhibitor approved by the U.S. Food and Drug Administration (FDA) for the treatment of adults and adolescents (≥16 years) with advanced or metastatic epithelioid sarcomas, which are not suitable for surgery or other standard treatments, and for relapsed/refractory follicular lymphoma in adult patients [31]. In recent decades, numerous EZH2 inhibitors have also been investigated as therapeutic options for head-and-neck tumors (Kidani et al. [33]; Idris et al. [38]).

Although numerous authors agree that high intratumoral EZH2 expression indicates poor prognosis in regard to HNSCC (Mochizuki et al. [34]; Chang et al. [35]; Wu et al. [36]), some controversy remains regarding which tumor characteristics (e.g., lymph node metastasis (Nienstedt et al. [21])) are the most significantly affected by the actions of EZH2.

Our results also indicate that differences might even exist between age and localization-based subgroups. In our study, the proportion of EZH2-expressing cells was the highest in the subpopulation with the worst prognosis (hypopharyngeal carcinoma patients) and the lowest in those with the best DFS rates (laryngeal carcinoma patients). For our young patients, linear regression analysis indicated only a possible marginal effect of EZH2 expression on patient survival, and the very low standardized R^2^ value suggests the relevance of further, likely multiple, factors, a result that is in line with our hypothesis. Cox analysis only identified advanced N and T statuses, as well as alcohol consumption, as significant negative prognostic factors for survival.

EZH2 might influence cisplatin resistance by promoting DNA repair through the upregulation of the expression of DNA damage repair genes. Combination therapy consisting of EZH2 inhibitors and cisplatin could potentially be beneficial for the treatment of lung, ovarian, and breast cancer. Synergistic effects were reported in two in vitro models; however, another study suggested there was an antagonistic effect in HNSCC cell lines [39]. Due to the immunomodulatory effects of EZH2 through MHC I down- and PD-1 up-regulation, the combination of EZH2 and checkpoint inhibitor therapies might enhance the therapeutic response [9].

In summary, although EZH2 is a well-established marker of tumor aggressivity, our results suggest that its expression is decreased and its prognostic role might be marginal in young HNSCC patients. Further studies might need to put more emphasis on patient characteristics beyond cellular carcinogenesis, as well as on a wider range of environmental factors and the fine-tuning of tumor immunity in young HNSCC patients. The EZH2 expression of malignant cells might also affect chemosensitivity; thus, researchers conducting pharmacological trials need to perform appropriate subgroup studies to investigate potential differences in the efficacy of EZH2 inhibitor therapies.

## 4. Patients and Methods

### 4.1. Patients and Study Setup

Formalin-fixed, paraffin-embedded tissue blocks of 68 young random HNSCC patients—diagnosed and treated between 2000 and 2018—were analyzed. We also involved the samples of 58 general HNSCC patients from the year 2014 as a control group. The general HNSCC group was matched for tumor localization and gender distribution. Patient data (age; gender; clinical history; tobacco and alcohol consumption; tumor site, stage, grade, and nodal status at the time of diagnosis; and time of death) were extracted from the hospital information system and the database of the Hungarian National Cancer Registry [40]. Consumers of alcoholic beverages at levels meeting or above the World Health Organization (WHO) medium-risk category (>40 g alcohol for men and >20 g for women) were considered drinkers, corresponding to the consumption of approximately a bottle of beer or >2–3 dL of wine on a daily basis [41]. Only those who had never smoked were considered non-smokers. All the formalin-fixed, paraffin-embedded tissue block samples originated from the archives of the Center of Tumor Pathology, National Institute of Oncology. This study was conducted under the ethical permission of the Scientific and Research Ethics Committee of the Medical Research Council (approval number: BMEÜ/3719- 1 2022/EKU) in accordance with the Helsinki Declaration of 1975, as revised in 2008.

### 4.2. Sample Processing and Evaluation

**Figure 5:**
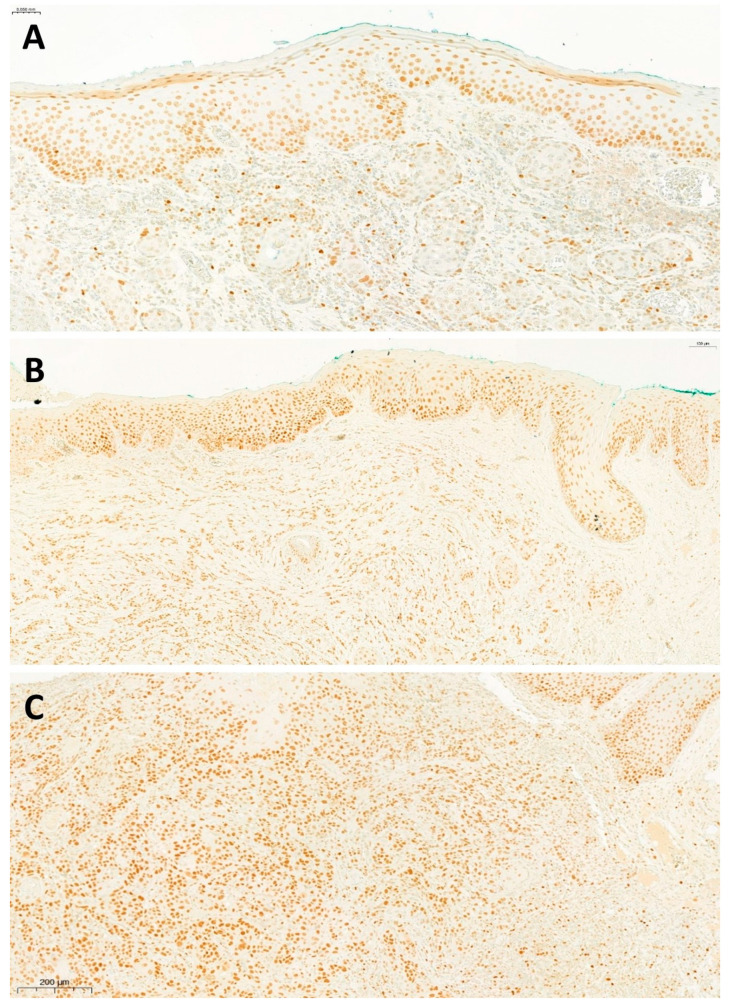
Representative samples and two different degrees of EZH2 expression (proportions of positive nuclei: (**A**) 20%; (**B**) 80%; (**C**) 100%). Tumor tissue can be seen in the submucosa; the dark tone in the nucleus indicates EZH2 expression. Positivity in the basal layer of physiological mucosal epithelium is normal. In the pictures, EZH2 expression extends to the full length of the epithelium due to the in situ carcinoma or epithelial dysplasia in the tumor environment. Scale bars represent 50 μm, on picture (**A**), 100 μm, on picture (**B**), 200 μm, on picture (**C**).

Hematoxylin-and-eosin-stained histological slides from the paraffin-embedded tissue sections were used to confirm diagnoses. All samples were taken before treatment. Immunohistochemical staining was performed to detect EZH2 expression (mouse monoclonal anti-EZH2 antibody, clone 11, BD Biosciences, Franklin Lakes, NJ, USA). Heat-induced antigen retrieval and staining procedures were performed according to the manufacturer’s instructions. The immunohistochemical reaction was visualized using the UltraView DAB kit (Ventana, Tucson, AZ, USA). Two independent examiners (AS, ET) evaluated the reactions and scored the staining intensity and the proportion of positive cells. Lymphocytes were used as negative controls and lymph node germinal centers were used as positive controls for the immunohistochemical staining for EZH2. The proportion of positive tumor cells (%) and the intensity of nuclear staining (0, 1+, 2+, 3+) were recorded for each slide. Representative samples of different degrees of EZH2 expression are shown in Figure 5. The expression levels of p53 protein were also examined by means of immunohistochemistry (mouse monoclonal anti-p53 antibody, clone DO7, DAKO, Glostrup, Denmark). For p53 staining intensity, the positive control was a monoclonal, p53-expressing, high-grade serosus ovarian cancer cell line. Negative nuclear staining corresponded to a score of 0, focal or heterogenous staining patterns corresponded to a score of 1, and diffuse intensive nuclear staining in ≥80% of the tumor cells was classified as a score of 2.

### 4.3. Statistical Analysis

Statistical analyses were conducted using KyPlot 5.0 (KyensLab Inc., Tokyo, Japan). Kolmogorov–Smirnov test was used to assess the distributions of numeric variables. *t*-test or Mann–Whitney U test (MWU) was used to compare independent variables according to distribution normality. Kruskal–Wallis test was used for non-parametric multiple comparisons, using Dunn’s test for post hoc analysis. Chi-square test (CS) was used to compare the compositions of groups. Overall survival intervals were determined as the time period from discovery date to the time of death in months. Survival data were processed using the Kaplan–Meier method, while the survival of subgroups was compared using the Log-Rank (Cox–Mantel–Haenszel) test. Univariate and multivariate analyses of prognostic factors were performed using the Cox’s regression model. A *p* value < 0.05 was accepted as statistically significant.

## Figures and Tables

**Table 1:** Clinical characteristics of the young patients and the control group.

	Young HNSCC	General HNSCC	*p*
Case number (n)	68	58	
Sex (female/male)	16/52	10/48	0.39 (χ^2^)
Age (median; min-max)	36 (21–39)	62 (45–88)	0.001 (MWU)
Smoking history pos neg NA	43 (63.2%) 22 (32.3%) 3 (4.4%)	46 (79.3%) 12 (20.7%) 0 (0%)	0.10 (χ^2^)
Alcohol consumption (n; % of group) pos neg NA	29 (42.6%) 36 (52.9%) 3 (4.4%)	37 (63.8%) 21 (36.2%) 0 (0%)	0.03 (χ^2^)
Localization (n; % of group) Lip, oral cavity Mesopharynx Hypopharynx Larynx	41 (60.2%) 13 (19.1%) 7 (10.3%) 7 (10.3%)	26 (44.8%) 10 (17.2%) 11 (18.9%) 11 (18.9%)	0.19 (χ^2^)
5 years DFS (yes/known) Total Lip, oral cavity Mesopharynx Hypopharynx Larynx	46.2% (30/65) 43.6% (17/39) 61.5% (8/13) 0% (0/7) 83.3% (5/6)	34.5% (20/58) * 30.8% (8/26) * 50% (5/10) * 18.2% (2/11) * 45.5% (5/11) *	0.18 (χ^2^) * 0.29 (χ^2^) * 0.58 (χ^2^) * 0.67 (χ^2^) * 0.11 (χ^2^) *
Primary tumor size (n) T1 T2 T3 T4	19 28 8 13	12 24 10 12	0.71 (χ^2^)
Nodal status (n) N0 N1 N2 N3	27 11 27 3	32 6 17 3	0.83 (χ^2^)
Stage (n) I II III IV	12 8 14 34	10 11 9 28	0.67 (χ^2^)
Grade (n) 1-2 3-4 NA	49 15 4	35 21 2	0.13 (χ^2^)

NA: not available. * Survival data are presented to characterize the study groups. Due to group matching for localization and gender, the data are not suitable for drawing epidemiological conclusions.

**Table 2:** Staining characteristics of tumor cells of the young patient and control HNSCC groups.

	Young Patients	Control	*p* (Stat. Method)
EZH2 expression (% of malignant cells) Total (multiple comparison) Lip, oral cavity Oropharynx Hypopharynx Larynx	median [IQR] 40 [3.75–72.5] - 40 [5–60] 20 [0–80] 60 [50–70] 10 [0–60]	median [IQR] 60 [30–80] - 60 [20–90] 60 [25–80] 80 [60–90] 40 [10–80]	0.003 (MWU) 0.01 (Kruskal–Wallis) 0.02 (Dunn post hoc) 0.20 (Dunn post hoc) 0.04 (Dunn post hoc) 0.08 (Dunn post hoc)
EZH2 expression (staining intensity *) Total (multiple comparison) Lip, oral cavity Oropharynx Hypopharynx Larynx	median [IQR] 1 [0.75–2] - 1 [1–2] 2 [0–2] 2 [1–2] 1 [0–1]	median [IQR] 1.5 [1–2] - 2 [1–3] 2 [1–2] 3 [2–3] 1 [1–2]	0.0001 (MWU) <0.001 (Kruskal–Wallis) 0.02 (Dunn post hoc) 0.16 (Dunn post hoc) 0.006 (Dunn post hoc) 0.02 (Dunn post hoc)
p53 expression (staining intensity *) Total (multiple comparison) Lip, oral cavity Oropharynx Hypopharynx Larynx	median [IQR] 1 [0–2] - 1 [0–2] 1 [0–1] 1.5 [0.75–2] 1 [0–1.25]	median [IQR] 1 [0–2] - 1.5 [0–2] 1 [0.5–2] 2 [0.5–2] 1.5 [0.5–2]	0.26 (MWU) 0.70 (Kruskal–Wallis) N/A N/A N/A N/A

N/A: not applicable. * Semi-quantitative score (0–3 for EZH2 and 0–2 for p53).

**Table 3:** Cox analysis for the predictors of patient survival among young HNSCC-afflicted individuals.

	Univariate		Multivariate	
	HR (95% CI)	*p*	HR (95% CI)	*p*
EZH2-expressing tumor cells (%, above vs. at or below median)	1.2 (0.63–2.29)	0.59	1.17 (0.54–2.51)	0.69
p53 expression (expressing vs. non-expressing)	0.9 (0.47–1.78)	0.80	0.8 (0.35–1.63)	0.47
T status (T3-4 vs. T1-2)	2.34 (1.23–4.46)	0.01	1.64 (0.78–3.47)	0.19
N status (N+ vs. N0)	2.92 (1.38–6.2)	<0.01	3.04 (1.38–6.68)	<0.01
Alcohol consumption (vs. below WHO medium risk)	2.26 (1.16–4.4)	0.02	1.96 (0.98–4.08)	0.06
Smoking (vs. non-smoker)	2.02 (0.92–4.44)	0.08	1.59 (0.65–3.94)	0.31

HR: hazard ratio; CI: confidence interval.

## Data Availability

All data used in the preparation of the paper are available on reasonable request.
